# High resolution ultrasound imaging for repeated measure of wound tissue morphometry, biomechanics and hemodynamics under fetal, adult and diabetic conditions

**DOI:** 10.1371/journal.pone.0241831

**Published:** 2020-11-23

**Authors:** Surya C. Gnyawali, Mithun Sinha, Mohamed S. El Masry, Brian Wulff, Subhadip Ghatak, Fidel Soto-Gonzalez, Traci A. Wilgus, Sashwati Roy, Chandan K. Sen

**Affiliations:** 1 Department of Surgery, Davis Heart and Lung Research Institute, Center for Regenerative Medicine & Cell-Based Therapies, The Ohio State University Wexner Medical Center, Columbus, OH, United States of America; 2 Department of Surgery, IUH Comprehensive Wound Center, Indiana Center for Regenerative Medicine and Engineering, Indiana University School of Medicine, Indianapolis, IN, United States of America; 3 Department of Plastic and Reconstructive Surgery, Zagazig University, Zagazig, Egypt; 4 Department of Pathology, The Ohio State University Wexner Medical Center, Columbus, OH, United States of America; University of New South Wales, AUSTRALIA

## Abstract

Non-invasive, repeated interrogation of the same wound is necessary to understand the tissue repair continuum. In this work, we sought to test the significance of non-invasive high-frequency high-resolution ultrasound technology for such interrogation. High-frequency high-resolution ultrasound imaging was employed to investigate wound healing under fetal and adult conditions. Quantitative tissue cellularity and elastic strain was obtained for visualization of unresolved inflammation using Vevo strain software. Hemodynamic properties of the blood flow in the artery supplying the wound-site were studied using color Doppler flow imaging. Non-invasive monitoring of fetal and adult wound healing provided unprecedented biomechanical and functional insight. Fetal wounds showed highly accelerated closure with transient perturbation of wound tissue cellularity. Fetal hemodynamics was unique in that sharp fall in arterial pulse pressure (APP) which was rapidly restored within 48h post-wounding. In adults, APP transiently increased post-wounding before returning to the pre-wounding levels by d10 post-wounding. The pattern of change in the elasticity of wound-edge tissue of diabetics was strikingly different. Severe strain acquired during the early inflammatory phase persisted with a slower recovery of elasticity compared to that of the non-diabetic group. Wound bed of adult diabetic mice (db/db) showed persistent hypercellularity compared to littermate controls (db/+) indicative of prolonged inflammation. Normal skin strain of db/+ and db/db were asynchronous. In db/db, severe strain acquired during the early inflammatory phase persisted with a slower recovery of elasticity compared to that of non-diabetics. This study showcases a versatile clinically relevant imaging platform suitable for real-time analyses of functional wound healing.

## Introduction

Non-invasive interrogation of the functional aspects of cutaneous wound healing is highly powerful in helping to understand the tissue repair continuum through repeated measurements of the same wound [1]. Onset of injury marks the beginning of wound healing which, through a number of inter-dependent mechanisms, advances as one continuous process [2]. Any interruption of such processes by invasive measures such as tissue biopsy may be viewed as a confounding factor that complicates the original cascade of event elicited by another injury on an already wounded tissue [3]. The study of different animals for different time points is useful within narrow limits, as individual variations in the kinetics of response to injury are likely. Thus, the significance of such study of wound pathology may be escalated to the next level by interpreting the findings in the context of non-invasive functional studies of the same wound. Development of novel non-invasive imaging modalities to study functional aspects of wound healing confers the additional power of offering a clear path to improve clinical standards of care [2]. Histological quantification of skin and its appendages has been accepted as a standard method to demarcate epidermal and dermal thickness [4]. Although such approach is powerful in discriminating cell populations within the skin based on morphological features, it must rely on invasive tissue biopsy interrupting the tissue repair process [4]. Additionally, post-harvest tissue processing, such as dehydration, introduces changes that depart from the natural state of the wound tissue. At a more macroscopic scale, imaging approaches may provide critically important functional data that may act as a temporal reference line to enhance histological data interpretation [2].

Current imaging modalities offer in-depth assessment of the wound bed and its surroundings [1, 5, 6]. The ability to directly visualize tissue dynamics with high spatiotemporal resolution in these small-animal models at the whole-body scale provides novel insights into biological processes at the whole-organism level [7]. In this context, the noninvasive ability to enable three-dimensional (3D) visualization of the structure as well as quantification of functional parameters of the wound bed provides information of significant clinical relevance. Current non-invasive imaging devices such as thermography, macrophotography, laser speckle perfusion mapping, and laser Doppler flowmetry are effective for noninvasive superficial wound mapping [1, 8]. Although these non-invasive methods are beneficial, these offer only superficial assessment and are dedicated only for vascular perfusion assessment. Ultrasound imaging technique on the other hand is a superior to these modalities in many aspects. Ultrasonography can assess anatomy, hemodynamics, and elastography which are the key factors to perform wound healing assessment non-invasively. In addition to these, ultrasound is a validated tool [9–11] to measure wound volume which is crucial to monitor the kinetics of wound healing over time. Identifying remodeling of the tissue and its scarring can be performed through the changes in tissue texture and granulation in ultrasound B-mode images and elastograms which proves this technique as powerful technique in wound healing assessments [12]. Ultrasonography techniques can quantify morphological parameters of biological tissue through measurements of acoustic propagation which depend on factors such as velocity, attenuation, absorption, and echogenic scattering from tissue [1, 9]. We and others have reported on the use of ultrasound imaging to characterize wound healing in a pre-clinical swine model [9, 12]. The use of harmonic ultrasound imaging afforded high resolution scrutiny of the superficial skin and mapping of tissue elasticity [13]. The power of ultrasound harmonicity allowed the mapping of tissue elasticity as a unique feature [9]. Ultrasound has been instrumental in monitoring fetal development and diagnosis of pre-natal disorders. Fetal complications such as pre-labor rupture of membranes, placental abruption, and chorioamnionitis can cause surgical risk [14] demanding a non-invasive procedure such as ultrasound imaging. Interventional intrauterine fetal surgeries often result in inflicting fetal wounds. Such wounds require non-invasive real time assessment. In this study, we show the first evidence for animal studies using ultrasound in monitoring fetal wound healing. In this work, we seek 3D visualization of deeper structures as a powerful approach to study full-thickness wounds [15]. In contrast to our previous work on harmonic ultrasound imaging [9], here we utilize a motorized platform aimed at holding and moving the ultrasound transducer across the region of interest. Image thus collected were reconstructed to generate 3D image or video as reported previously [15]. The display of such information in the cross-sectional, sagittal or coronal planes provides the power of 360° visualization of the region of interest. Furthermore, we harness the power of simultaneous multi-mode high resolution ultrasound imaging where data are collected for B-mode (anatomy and elastography) as well as color Doppler flowmetry [15–17]. The major constituents of cutaneous extracellular matrix proteins mainly collagen and elastin contribute to biomechanical properties of the skin. They play major role in stiffness and elasticity of skin respectively. Collagen in wound healing forms a mesh-like scaffold in the damaged tissue and seals off the wound by forming a scar [18–20]. In fetal skin the extracellular matrix composition is different as compared to the adult skin, such as the collagen I to collagen III ratio [21, 22]. Elastin is not present in the dermis of the fetal skin. However, it is found in the adult skin. Further, the expression pattern of several epidermal proteins such as involucrin, basement membrane proteins, chondroitin sulfate, are higher. The expression patterns of basement membrane proteins, blood vessels, K10, K14, K16 and epidermal Ki-67 are similar in fetal skin and adult skin. These differences play a role in regenerative phenotype of the fetal wound healing [23]. Fetal wound healing compared to adult wound healing has been shown to have a different and reduced inflammatory response. Also the presence of inflammatory cells is short lived in fetal wound healing compared to the adult [24, 25]. These established facts warrant the study of the differences in healing of fetal and adult wounds.

This work reports first evidence of using such imaging in cutaneous wound healing. In addition, biomechanical characterization of the healing dynamics for fetal, adult and diabetic adult, along with a comparison between diabetic and normal adult wound healing was performed. It is established that fetal wound healing is rapid regenerative which is lacking in adult [25] cutaneous wound healing. On the other hand, fetal circulation is governed by placental blood flow which provides oxygen and nutrients to the fetus via the umbilical artery. The highest oxygen saturation of this blood is 80% compared to 100% in healthy adults [26]. There are blood and oxygen circulation differences and cardiac function in the fetus compared to the neonates and adults. As an example, the inotropic ability of the fetal and neonatal heart is not identical [27]. Thus, due to the fetal environment in womb, these differences in circulation may lead to the faster recovery of the pulse pressure in fetuses than in adults.

As an application of the technique, we performed fetal wound healing assessment to validate its application in small animal cutaneous wound healing which is known to be highly efficient [28, 29] while diabetic wound healing in the adult is widely acknowledged to be complicated [1].

This work provides an account of the biomechanical characterization of the healing dynamics for fetal, adult and diabetic adult, along with a comparison between diabetic and normal adult wound healing.

Furthermore, morphometric studies examining wound closure are supported by the study of tissue elasticity and blood flow leading to novel hypotheses [30]. Kinetics of intrauterine fetal wound healing revealed highly accelerated closure. Non-invasive monitoring of fetal and adult wound healing provided unprecedented biomechanical and functional insight. This study showcases a versatile clinically relevant imaging platform suitable for real-time analyses of functional wound healing.

## Materials and methods

### Animals

All animal studies were approved by the Ohio State University Institutional Laboratory Animal Care and Use Committee (ILACUC) under protocol 2009A0214-R2. All methods were performed in accordance with the relevant guidelines and regulations to this effect.

#### Pregnant mother and fetus

Timed pregnant FVB/NJ mice (Taconic Biosciences; Hudson, NY, USA) were housed in individual cages at the vivarium and fed food and water *ad libitum*. A total of six pregnant mice at a gestational age were used. Mice homozygous (BKS.Cg-m+/+Lepr db/J 482, or db/db; stock no 000642) for spontaneous mutation of the leptin receptor (Leprdb) (aged 8–10 weeks) and heterozygous littermate control db/+ [26] were obtained from Jackson Laboratory, Bar Harbor, ME. All the fetal surgeries were done on pregnant female FVB mice. Maternal heart rate and rectal temperature were monitored and heating was adjusted to maintain rectal temperature close to 37.5°C. All hair was removed from the abdomen by shaving, followed by a chemical nair and cleaned with alcohol swab. Mice were lightly anesthetized with 1.5% isoflurane in oxygen during ultrasound exams. The application of isoflurane as an inhalational anesthetic in the mouse was optimized to attain stable hemodynamics by administering it at 1.5% by-volume. The concentration of isoflurane was kept ≤1.5% to keep its’ side effect minimal. Although the heart rate was lowered due to isoflurane affecting the flow/pulsation, this is standard in other studies as well such as cardiac function in mice [31]. Mice were housed under a 12 h light-dark cycle with food and water *ad libitum*. The animals were tagged and grouped randomly 5–6 animals in each group.

### Fetal cutaneous wound model

FVB/NJ (8 wk old) female mice were used. Full thickness incisional wounds were generated in utero on embryonic day E15.5 as described previously [32] on three different dams. Briefly, a laparotomy was performed under aseptic conditions on time-mated FVB Mice (Taconic Biosciences; Hudson, NY, USA). Two-millimeter full thickness incision was made in the dorsal skin of fetus, using microsurgical scissors. One microliter of phosphate buffered saline containing 10% India ink (Becton Dickinson; Sparks, MD, USA) was injected subcutaneously at the wound site for subsequent wound identification. During the ultrasound imaging, the dorsal side of the fetus was scanned. The scattered or reflected ultrasound echoes were collected as an image signal. Based on the difference in acoustic impedance of different tissues the tissue types were identified. Wounds were with low echogenicity than normal tissue in ultrasound B-mode images [6]. The fetuses were euthanized for wound tissue collection at 48h post-wounding. All animal experiments were carried out with approval from Institutional Animal Care and Use Committee, The Ohio State University.

### Adult cutaneous wound model

B-mode imaging was performed on adult normal healthy (n = 3 each group) mice. Two 8 mm punch biopsy excisional wounds were created on the dorsum skin of control (db/+, n = 6) and diabetic (db/db, n = 6) mice (Jackson Laboratory, Bar Harbor, ME) equidistant from the midline and to avoid purse-string contractions, we applied stents which resulted in a uniform healing via epithelization thereby allowing wounds to heal through granulation and re-epithelialization. During the wounding procedure, mice were anesthetized by low-dose isoflurane inhalation as per standard recommendation. Wounds from age-matched db/+ and db/db animals were allowed to heal naturally.

### In vivo ultrasound imaging and processing

#### Data acquisition

A high-frequency ultrasound imaging system (Vevo 2100; Visual Sonics, Toronto, Canada) was used. Pre-warmed FDA approved completely aqueous, non-sensitizing and non-irritating ultrasound coupling gel (Aquasonic 100, Parker Laboratories, NJ) was used to avoid air gap for sound propagation. A 40 MHz high frequency ultrasound transducer (Vevo 2100; Visual Sonics, Toronto, Canada) was positioned on the abdomen of the mice. B-mode imaging protocol was performed on the abdomen of pregnant mother until fetal anatomical location was visible clearly. In Doppler mode, the repetition frequency was set between 5 and 40 kHz to detect a range of low to high blood flow velocities. The angle between the Doppler beam and the vessel was maintained less than 15°. Waveforms were saved for later offline analysis. Heart rates of mother were measured from Doppler waveforms obtained from the heart and the uterine artery near the lateral-inferior margin of the uterocervical junction close to the iliac artery on each side [11, 17]. The image resolution was maintained at 100 μm laterally and 50 μm axially.

#### Fetal ultrasound imaging

B-mode mapping was performed on the abdomen of pregnant mother until fetal anatomical location was visible clearly. During ultrasound imaging, the pregnant mice were anesthetized with a mixture of 1.5% isoflurane in carboxin by inhalation through mouse nose-cone. The pregnant mothers’ body temperature was kept at 37°C during the experiment with a temperature-regulated platform. From each of the six pregnant mice, two fetuses with the most favorable spatial orientation (either left or right lateral position) in the lower abdomen were chosen for imaging the regions of interest. Each targeted fetus was visualized in its longitudinal axis using two-dimensional (2D) imaging for the visualization of fetus and the wound. Fine position adjustment was done for the highest signal intensity with continuous adjustment of contrast, dynamic range, and gain until the minimal noise appeared in the background. For wound volume measurement, a 3D-Mode of Ultrasound imaging was used to collect three-dimensional data. For this part of acquisitions, a set of motorized unit (Vevo 2100; Visual Sonics, Toronto, Canada) was connected with the Ultrasound transducer and the main Ultrasound system. The probe and the translation motor were attached orthogonal to each other and the imaging plane which moves across the target fetus in a series of small steps with 3D step size: ranged 0.04 mm—0.05 mm. Translation of the probe-motor system was set such that it covered the abdomen of the pregnant mice and of course fetuses of interest. From this translation, B-mode 3D recording clips of each up to 50 s length were saved. After collection, the 3D data transferred to workstation computer, the images/video was read off-line and semi-automated tracing the boarders of the wounds were performed. In the semi-automated tracing process, the auto traced frames were smooth and the one manually traced were pixelated which can be observed in Fig 2B. The wound volume was then automatically computed using Simson’s’ rule. Color Doppler flowmetry of fetal heart, and wound feeder vessel flow were also recorded using color Doppler flow imaging (CFI) mode both in 2D and 3D modes.

#### Adult ultrasound imaging

Normal healthy adult db+/ and db/db mice (n = 3) underwent ultrasound B-mode imaging for healthy skin data. B-mode imaging was performed on the dorsum of adult mice until anatomical location of wounds was visible clearly. During ultrasound imaging, the mice were anesthetized with a mixture of 1.5% isoflurane in carboxin by inhalation through mouse nose-cone and body temperature was kept at 37°C on a temperature-regulated platform. Using Vevo 2100 ultrasound machine the following parameter values were used. Image depth: 12 mm from skin surface, signal gain: acquisition gain range of 20 dB—22 dB, persistence: Off, line density: high, dynamic range: 65 dB, 3D step size: ranged 0.04 mm—0.05 mm, and sensitivity: high.

#### Image processing

The image processing and strain analyses that is applicable to both fetal and adult tissue are provided through flow charts as a S1A Fig in S1 File. The wound volume was measured during post-processing. VevoLab® software (Visual Sonics, Toronto, Canada) was used to compile each 2D image slice with other acquired slices and rendered them into a 3D reconstruction resulting into a volumetric object. From the B-mode recordings, the frame-wise tracing of wound edge borders was performed semi-automatically using three-dimensional (3D) volume processing option in the software which enables to measure lengths, areas, and volumes. The tracing was performed manually in different frames of the 3D recorded video. The algorithm in package then automatically traces the wound boarders on the frames that are in between the manually traced frames. In manual tracing a ‘+’ mark appears on the pointer arrow. Since the points of selection are so close each other these may appear crowded. In automated tracing that doesn’t happen. The 3D-mode provides a three-dimensional view of an area of interest from frame-based imaging. The 3D visualization of wound videos was also recorded. Wound depth and diameter were measured from all central frames recorded and the volumes were computed using wound area (A = πD^2^/4) multiplied by the depth (d). Where D = diameter and d = depth.

### Fetal wound tissue strain analysis

By definition the ratio of the change in length to original length is strain. Soft tissue strain is defined by the equation,

$$\varepsilon =\frac{\delta }{L}$$ (1)

where δ is change in length called elongation, L is initial length which provides information about the mechanical properties of tissues, such as their hardness or stiffness. Tissue strain measurement using ultrasonography is an emerging technology. Ultrasonic estimation of soft tissue strain is generally more accurate and precise, it is relatively fast and more convenient in measurements [13]. In this study, tissue strain was analyzed by ‘Vevo Strain’ package and the pre- and post-compression of ultrasound echo signals with the technique called speckle tracking, which actually measures the tissue motion under an ultrasonic compression and relaxation pressure. Ultrasound B-mode video recording was performed on the fetuses at 3, 24, and 48h post-wounding. Unwounded skin and wound tissue elasticity measurements were performed using ‘Vevo Strain’ software. To do so on a time-frame of a B-mode video clip, a green U-shaped curved line starting from skin tissue and passing through wound bed and edge was drawn which allowed us selecting points of interest to measure corresponding tissue strain. By hitting ‘Start analysis’ icon, multiple windows such as B-mode video, the axial strain, the longitudinal strain, and a 3D color image representing the strain data over time appeared. Tissue of interest was selected by hoovering on the color 3D area, a point (blue, light green or red) on the green curve in the B-mode video appeared. This allowed to appear two strain curves on the longitudinal strain area corresponding to two points selected on the tissue of interest. Thus, the strain data so obtained were exported as a spread sheet file. Further processing was done in excel. Three points, one at each skin tissue sites (normal skin, wound bed and wound edge) on were selected for strain analysis of respective points. The strain measurement of the fetal skin, for the wound edge was done at 3h, 12h and 48h post-wounding whereas that for the wound bed was done at 12h and 48h. The 3h time point was avoided for wound bed due to presence of a void that resulted in the Ultrasound waves penetrating through underlying tissue. Because this provides reading which are not related to fetal skin strain.

### Adult wound tissue strain analysis

B-mode imaging was performed on adult normal healthy mice (n = 3 each group) and wounded db+/ (n = 6) and db/db (n = 6) mice. On a time-frame of B-mode video clip, a green U-shaped curve starting from wound edge tissue and passing through wound bed was drawn which allowed us selecting sites of interest using ‘Vevo Strain’ software package as mentioned above. Ultrasound guided analysis of biomechanical strain of adult normal skin, wound bed, and wound edge tissue were computed using the Vevo Strain® software. One point from each site (normal skin, wound-edge, and wound-bed) were selected for strain analysis. The software then provided strain data of the corresponding selected points over the entire video frames. The method of processing was as in the fetal strain analysis above.

### Adult wound tissue cellularity analysis

For cellularity, a Matlab code was written and used to obtain the signal intensity at the region selected. The signal intensity between the regions of interest (marked by the yellow arrows, representing the wound perimeter) was obtained. As more sound wave reflections occur from the region of high cellular density the image signal intensity was observed higher in the higher cell density regions. This enabled to measure tissue cellularity. Only wound bed area over time points was selected and compared. The entire image area was not used for analysis. As the wound healed, the ROI shrank.

#### Matlab code writing

A Matlab (Mathworks Inc. Natick, MA, USA) program was written as was done previously [8]. Gray scale images from ultrasound imaging system were transferred to Joint Photographic Experts Group (JPEG) image format. The JPEG images were processed to get color images which were used for the detection of cell density and granulated wound tissue both in fetal, adult normal skin and wounds. Equal area regions of interest (ROIs) were traced covering the wound site, fixed image depth and from the area signal intensity was computed.

### Histology of fetal and adult wounds

Pregnant female mice were humanely euthanized CO_2_ followed by cervical dislocation at E15.5 (0h post-surgery) and E18.5 (48h post-surgery) days of gestation. A surgical laparotomy was performed, and the uterine horn was removed and placed into ice-cold tissue collection buffer. The intact uterine horn was pinned out. Individual pups were identified, dissected free from surrounding tissues, and placed into a sampling dish; tissues were kept moist with phosphate buffer solution (PBS). The tissue was embedded in OCT compound in liquid Nitrogen Or processed as paraffin-embedded tissues. Briefly, paraffin-embedded adult tissues were cut into 8-μm thick sections. After deparaffinization and hydration, sections were stained with hematoxylin and eosin. Cryosectioned fetal samples of 10 microns were collected on positively charged plus microscope slides (Thermo Fischer) slides followed by hematoxylin and eosin (H&E) staining. For H& E staining, the tissues were stained with hematoxylin solution and were then rinsed in tap water until the water was colorless. The section was then counterstained with Eosin Y solution, dehydration with 95% ethanol. The section was then treated with Xylene. Mounting media was added and a coverslip was mounted for imaging. The mounting media was allowed to dry and the slide was imaged using Zeiss Axioscanner microscope in bright filed mode.

Immunostaining of K14 (Covance, PRb-155-100, rabbit anti-mouse, 1:400) was performed from OCT embedded tissues or paraffin embedded tissue [33]. Briefly, OCT embedded tissues were cryosectioned at 8–10 μm thick, deparaffinization followed by hydration was done, antigen retrieval was performed using sodium citrate (pH 6.0). Sections were, blocked with 10% normal goat serum and incubated with K14 antibody overnight at 4°C. Signal was visualized by subsequent incubation with fluorescence-tagged appropriate secondary antibody (Alexa 488-tagged α-rabbit, 1:200). Imaging was performed using Zeiss Axioscanner microscope in fluorescence mode.

### Pulse wave doppler velocity measurement and computation of pulse pressure

In the wounds the blood is supplied through feeder arteries. In the dorsal anatomy where wound was induced in this study, the feeder vessels are similar in size. In addition, the Doppler flow images provided arterial size measurement relatively precisely. This was the basis of identification of feeder vessels in adult wound [9]. In case of mice uterine arteries supplying oxygen and nutrition to the fetus, the runt pup(s) were avoided, male or female was not considered, however, the artery size (diameter) was maintained so that the chances of the fetuses to be alike can be maintained [34]. The pulse wave color Doppler feature of Vevo2100 Ultrasound system generated the blood flow velocity from the color flow images. Flow velocity profiles were obtained using Doppler flow mode of the Ultrasound protocol and pulse wave videos/images were analyzed from the pulse velocity profiles so obtained. From the images so obtained, the velocity of blood flow at systole and diastole was measured on all images both from fetal and adult feeder arteries. From the real-time velocity profiles to measure systole (profile peaks) and diastole (profile troughs) were obtained. Vessel diameters were measured to identify similar blood vessels in the desired area for all time points to maintain consistency. Velocity was calculated from three peaks and troughs. **P**(*v*), pulse pressure of the feeder artery as a function of velocity was measured using the modified Bernoulli equation,

$$P(v)=\frac{1}{2}\rho (v_{s}^{2}-v_{d}^{2})$$ (2)

where ρ is the density of blood, *v*_*s*_ the velocity at systole and *v*_*d*_ is the velocity at diastole.

### Statistical analysis

The data analysis was performed using student's t-test (two-tailed) to determine significant differences. Mean, standard error and student paired t-test analysis. Level of significance was considered to be 0.05. Data expressed as mean ± SE as reported in figure legends. No statistical method was used to predetermine the sample size. Power analysis was not necessary for this study.

## Results

### Fetal wound healing

**Fig 1:**
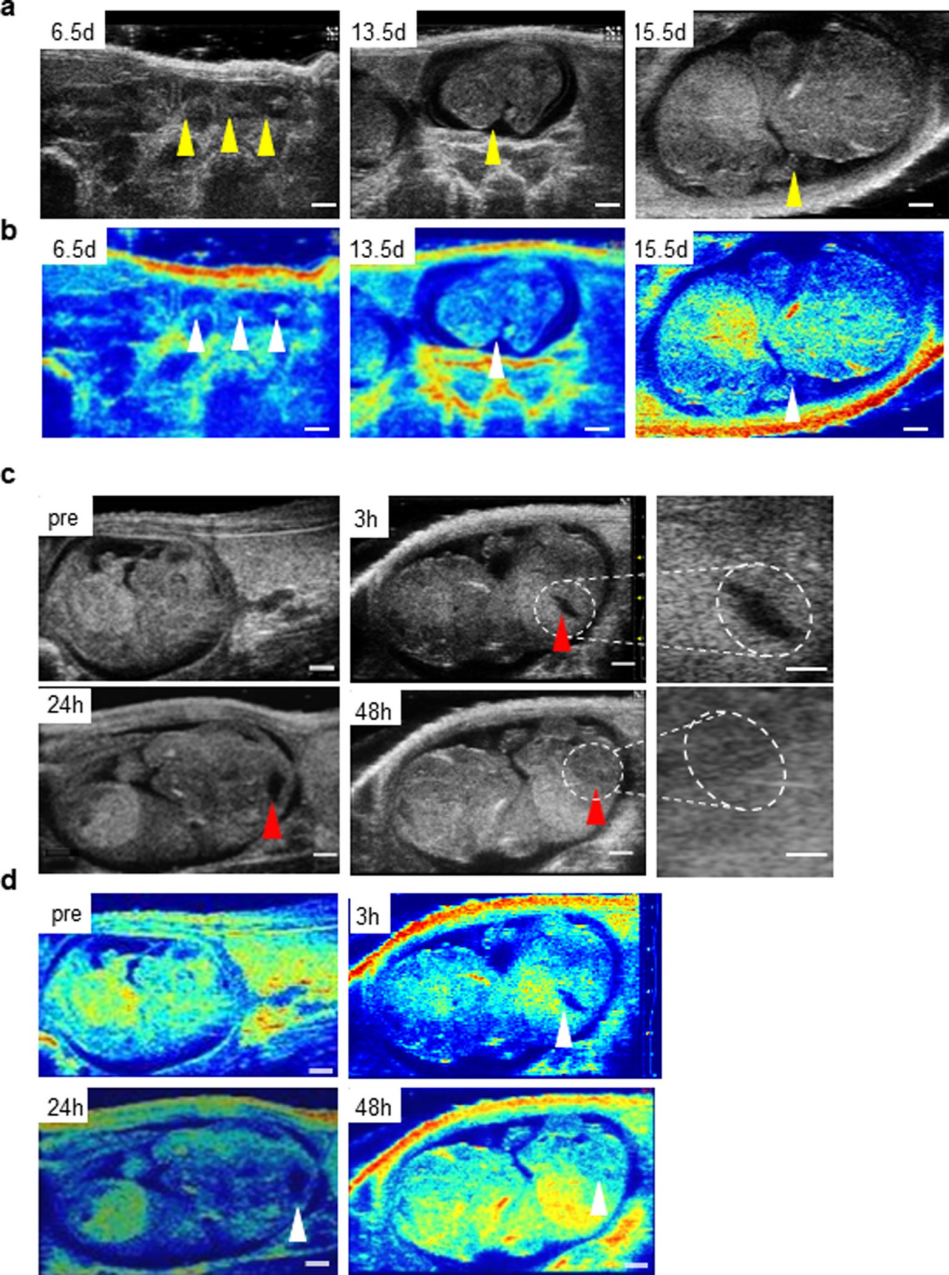
Non-invasive detection of early stage of fetuses and enhanced visualization of fetal wound tissue. (**a**) Ultrasound images of embryos at days E6.5, E13.5, and E15.5 (**b**) corresponding color-coded images for visual enhancement of tissue density *in vivo*, fetal positions shown by arrows. Wound-site marked with red arrow. Scale bar = 5 mm (c) Image panel showing fetal wound healing over time. Pre-wounding and post-wounding (3, 24, and 48h) (**d**) corresponding color-coded images are shown. Zoomed image of wounds at 3 and 48h post-wounding are marked by dotted circles as shown. Scale bar = 500 μm, n = 3. Wound-site marked with red arrow.

Ultrasound imaging may detect murine fetuses as early as on E6.5 days. Both anatomical structures as well as functional parameters are traceable. B-mode images identified the location where the embryos were formed (**Fig 1A**). Appropriate tuning of point density, contrast and 2D gain during data acquisition enabled the visualization of anatomy of fetal structures. The lateral perspective of three fetuses at E6.5 *in utero* was captured (**S1A Fig in S1 File and S1 Video)**. By E13.5, developed organs such as beating heart were detected (**S1 Video and S1A Fig in S1 File**). Development of Matlab™ code **(S1C Fig in S1 File**) and color coding (**Fig 1B**) were done to improve signal-to-noise ratio of B-mode images and clarity of visualization respectively as described in flowchart **(S1B Fig in S1 File)**. These are the enhancement of the gray scale signal intensity of the images in Fig 1A. Blue corresponds to low signal and red as higher signal intensity. Fetal surgical wounding was performed at E15.5 (**Fig 1C and 1D**). Because these wounds closed in 48h of injury, early time-points 3, 24, and 48 hours were studied. Zoomed images, demarcated by dashed lines, are presented for 3h and 48h time-points to contrast an open and closed wound, respectively (**Fig 1C**), (**S1A Fig in S1 File and S1 Video)**.

### Three-dimensional reconstruction of fetal wound tissue

**Fig 2:**
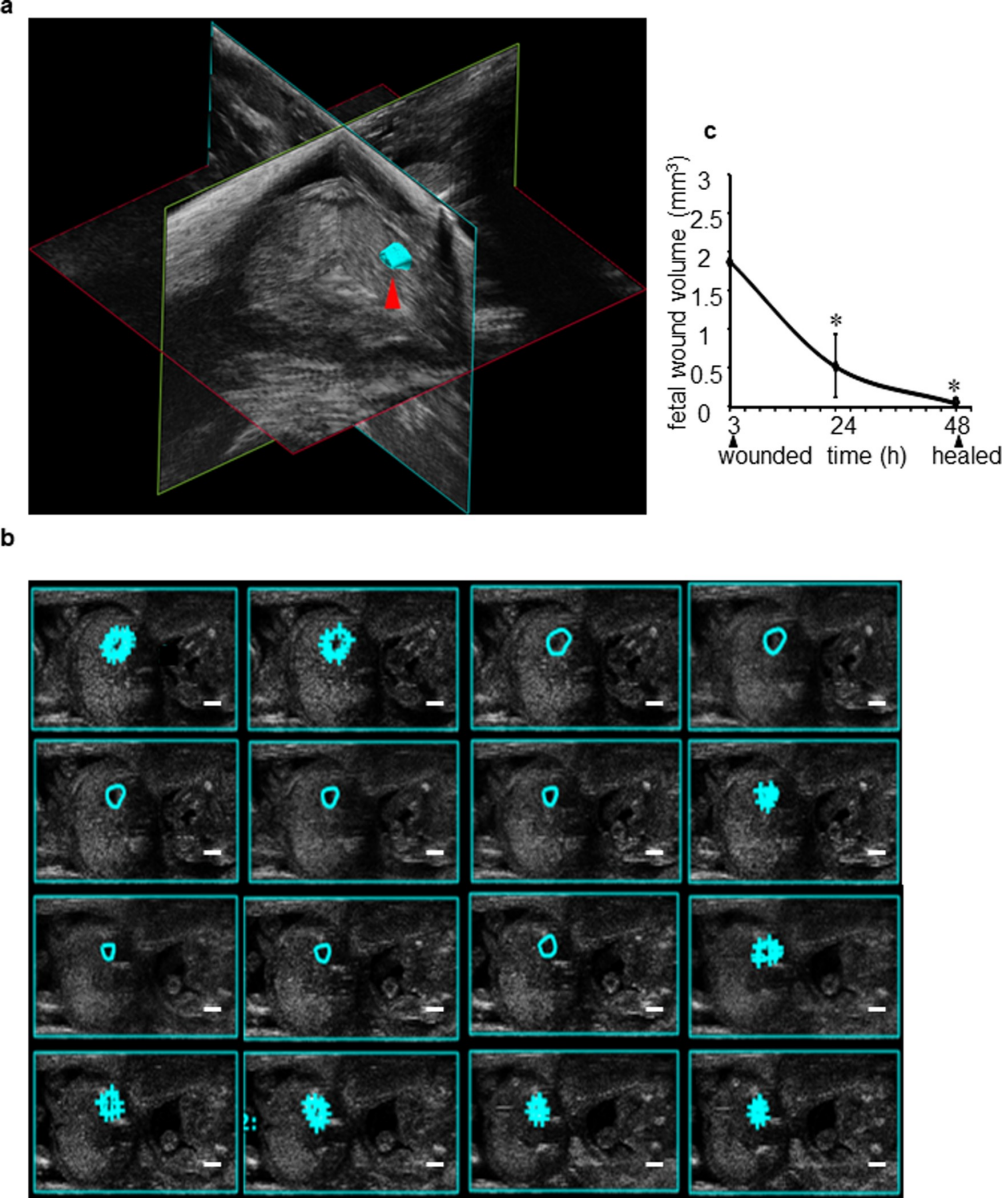
Volumetric fetal wound measurement using three-dimensional (3D) reconstruction of ultrasound images. (**a**) A 3D view of fetal wound (cyan) surface rendered 3D object constructed from 16 contours structure of fetal wound using the ultrasound B-mode images as shown by red arrow. (**b**) Semi-automated tracing of fetal wound border through different frames obtained from 3D visualization to measure wound volume (cyan). The tracing was performed manually in different frames of the 3D recorded video. The algorithm in ‘VevoStrain’ package then automatically traced the wound boarders on the frames that are in between the manually traced frames. (**c**) Line graph plot showing change in fetal wound volume over time at 3, 24, and 48h post-wounding. Data represented as the mean ± SE, n = 3 wounds. *p< 0.05.

A 3D-Mode of Ultrasound was used to collect three-dimensional data. For this part of acquisitions, a set of motorized unit (Vevo 2100; Visual Sonics, Toronto, Canada) was connected with the Ultrasound transducer and the main Ultrasound unit. This motor held and carried the Ultrasound transducer during its translation across the abdomen of pregnant mouse. Such path of the transducer enabled display of the surface rendered 3D object constructed from 16 contours structure of fetal wound (**Fig 2A**). To address challenges posed by the constantly changing orientation of live fetus, the transducer angle was adjusted. Recordings were post-processed to render and reconstruct a 3D view of the entire fetus. Three-dimensional-reconstruction enabled visualization in horizontal, sagittal, and coronal planes to characterize the wound parameters. A 3D localization of wound tissue was observed as shown in **Fig 2A and S2A Fig in S1 File**. Tracing of wound edges in the first frame, central frame, and the last frame of the main acquired video enabled the 3D processing function of the software to trace 16 frames resulting in the estimation of 3D wound volume (**Fig 2B**). Compared to the initial record at 3h post-wounding, wound volume sharply diminished at 24h and completely closed by 48h (**Fig 2C and S2B–S2D Fig in S1 File and S2A–S2C Video in S1 Video**).

### Histological characterization of fetal wound tissue

**Fig 3:**
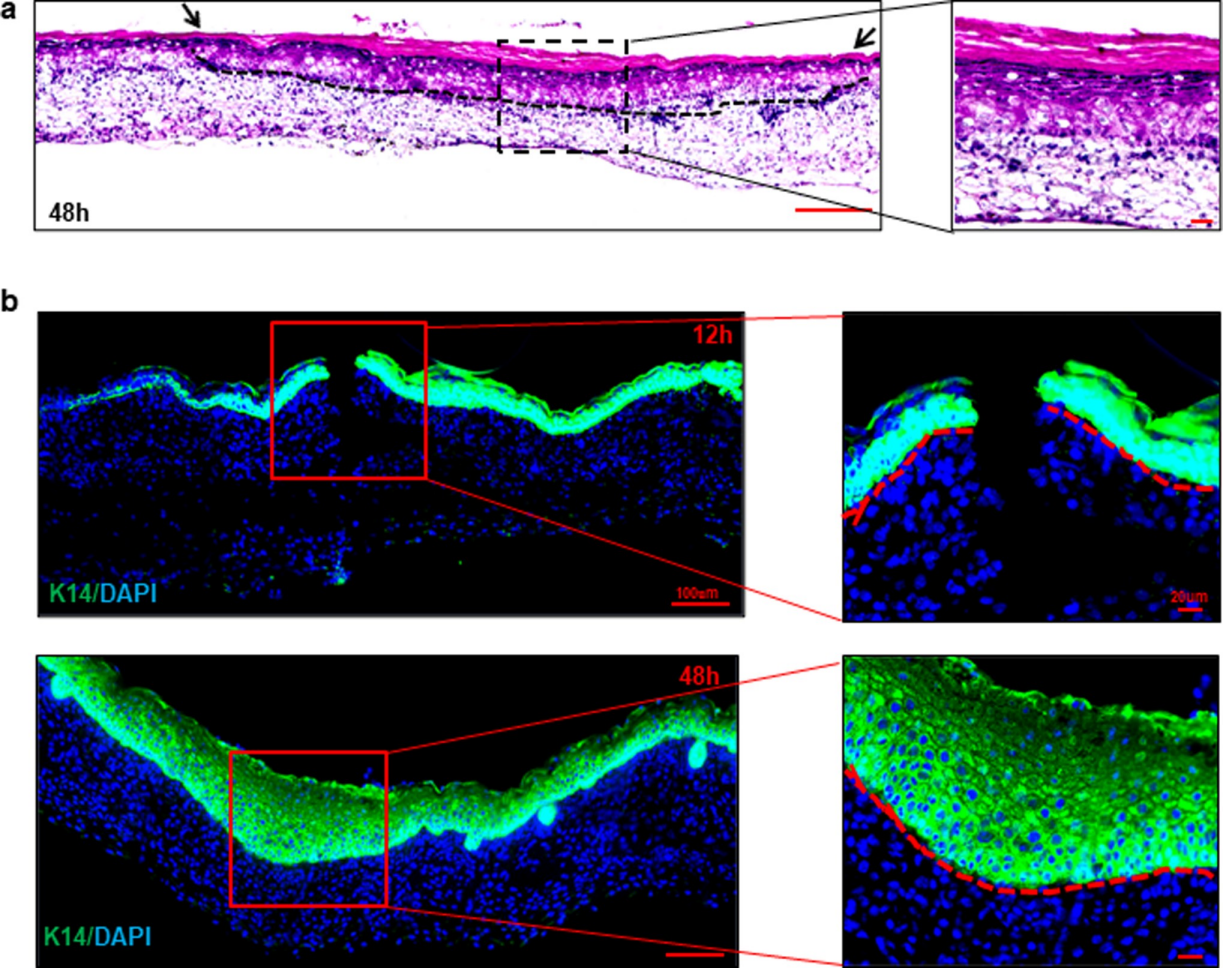
Histological characterization of fetal wound tissue depicted the re-epithelialization and vascularization of healing tissue. (**a**) Microscopic image of hematoxylin and eosin (H&E) stained fetal wound tissue obtained at 48h post-wounding (at E17.5 post embryo formation), wound site is marked by dotted line and wound edge is marked by arrows (upper panel). Scale bar = 200 μm. Zoomed images from the wound edge and wound bed (lower panel). Scale bar = 20 μm. (**b**) Immunohistochemical staining of keratinocytes (Keratin 14, green) and nucleus of cell (DAPI, blue) of the fetal wound tissue obtained at 12 h and 48h post-wounding (upper panel), (Scale bar = 100 μm). Zoomed images (Scale bar = 20 μm). The break in the epithelium at 12 h panel and hyperproliferative epithelium at 48 h panel represented the site of wounding respectively.

With the intent of histological validation of the reported imaging, fetal wound tissue was obtained at 12h and 48h post-wounding from the wound site. Hematoxylin and eosin (H&E) staining demonstrated wound closure at 48h (**Fig 3A**). Immunohistochemical localization using molecular markers of keratinocytes (Keratin 14) at 12h and 48h showed complete re-epithelization at 48 h (**Fig 3B**). These findings were consistent with imaging observations.

### Biomechanical property of fetal cutaneous wound site

**Fig 4:**
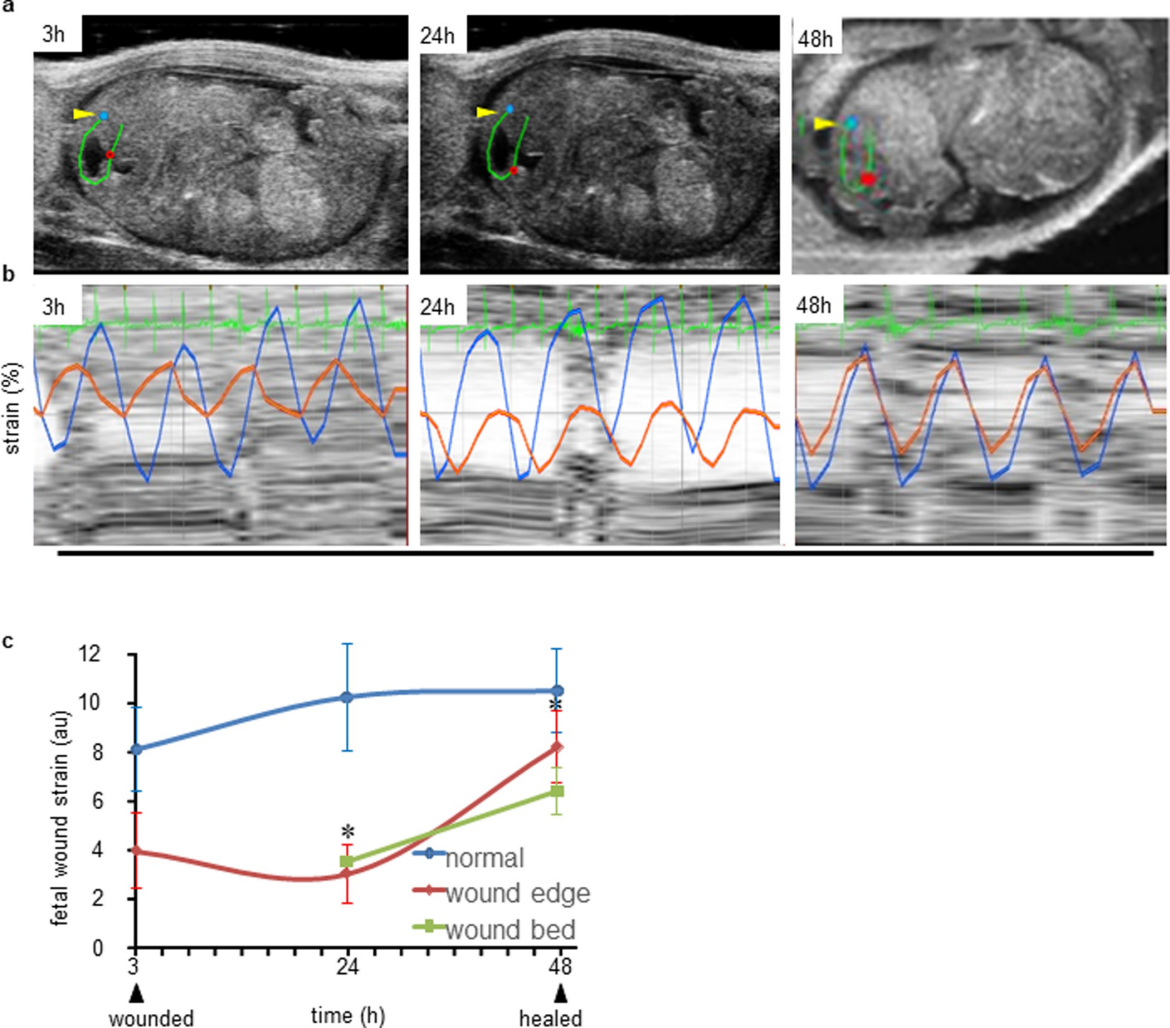
Noninvasive measurement of fetal cutaneous wound tissue stiffness. (**a**) Region selected for fetal wound edge (red dot) and surrounding un-wounded skin (blue dot) to analyze biomechanical strain. A line was traced (green) around the wound site to help select the analysis sites using ‘Vevo Strain®’ software. (**b**) Strain curves of edge (red) and surrounding unwounded skin (blue) at different time intervals (3, 24 & 48h) post-wounding. (**c**) Fetal wound strain of wound edge, wound bed and normal skin was computed from the red, light green and blue curves respectively and the line graph was plotted over time (3h-48h for normal skin and wound edge and 24h-48h for wound bed). The standard error of mean of wound bed at time points 24h, and 48h are 0.001, and 0.001) respectively. Data represented as the mean ± SE, n = 3. *p< 0.05.

For non-invasive strain measurement, B-mode recordings of fetal skin and wounds were performed at different time points (**Fig 4A**). Strain curves for fetal wound edge (red) relative to adjacent normal skin (blue) were asynchronous at 3h post-wounding (**Fig 4B**). Synchronicity in strain curves improved at 24h. Marked synchronicity was established at 48h supporting efficient tissue repair (**Fig 4B**). The accuracy of strain calculation, as provided by the software, was in the range of ± 0.6% to ± 1.34% post-wounding. The mean strain calculated from three fetal wound edges was significantly lower in 24h post-wounding than pair-matched unwounded fetal skin tissue. Wound bed tissue strain at 3h was higher than the wound edge tissue closer to the normal skin indicating that the sound propagates through the void at this time point indicating the strain of the internal structures (data not shown) and was as low as the wound edge in 24h which remained low even in 48h indicating wound bed as newer tissue (**Fig 4C**). Lower fetal wound edge tissue strain indicated higher tissue stiffness compared to the adjacent unwounded skin on the same fetus [5, 13] (**Fig 4C**). The wound bed strain data at 3h was measured but not shown here because this data is from internal structures not from the skin. However, it is presented from 24h-48h as new skin was generated. The strain is primarily contributed *via* the collagen and elastin present in the extracellular matrix and less by the other cellular constituents like keratinocytes [35, 36]. Hence, despite increase in keratinocyte layer not much appreciable increase in the strain value was observed.

### Adult murine cutaneous wound healing

**Fig 5:**
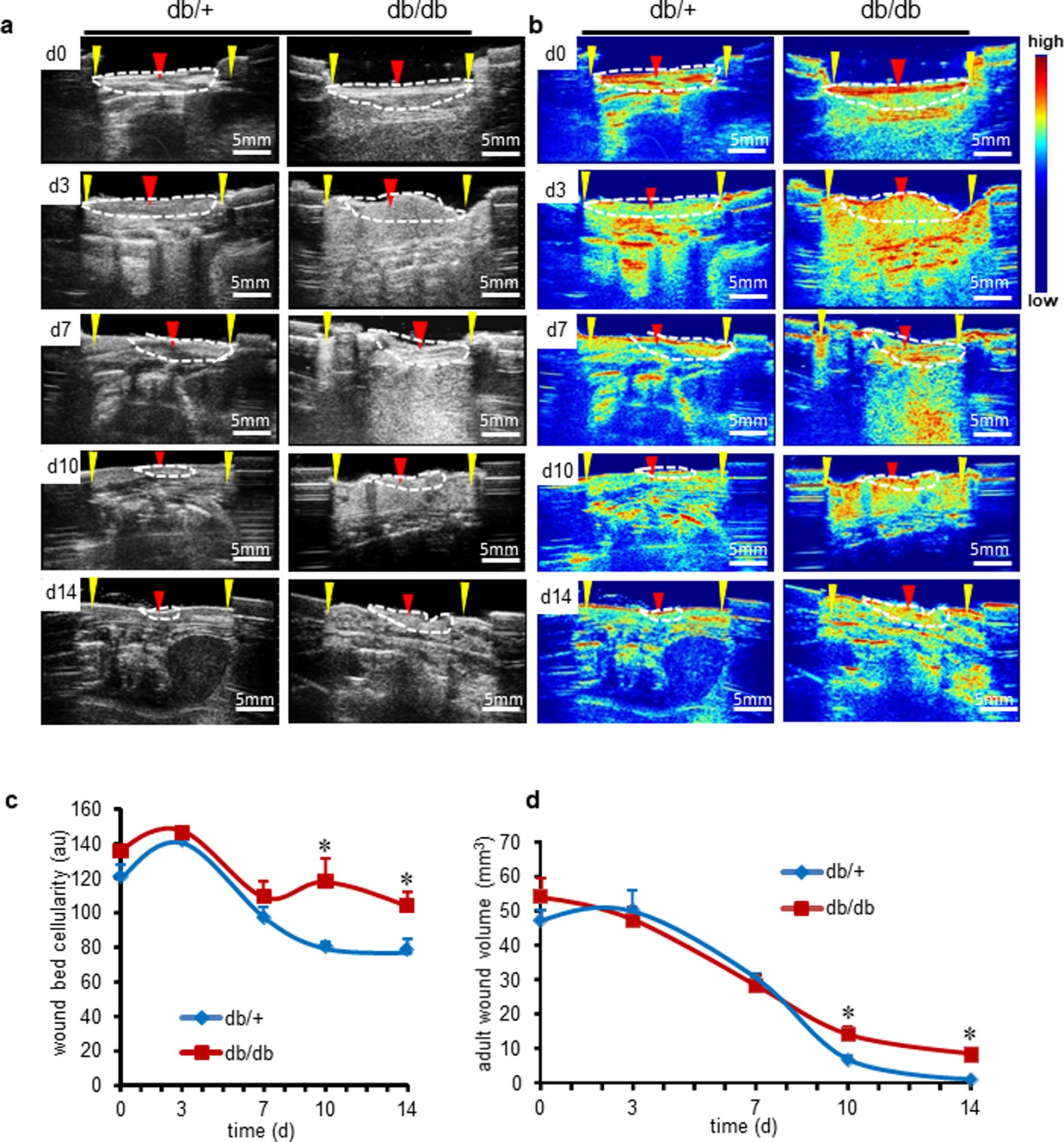
Morphometry of adult diabetic cutaneous wound healing compared to the non-diabetic mice. (**a**) Ultrasound B-mode image of full thickness stented wounds (d0,3,7,10,14) wound bed (red arrow, white dotted region), wound edge (yellow arrows) of db/db and control db/+ mice (**b**) Enhanced anatomical images using Matlab code to show the cellular density. Scale bar = 5 mm. Density index low (blue) to high (red) (**c**) Quantification of wound bed tissue cellularity over time in db/db and corresponding db/+ control. Data represented as the mean ± SE, n = 6. *p< 0.05. (**d**) Quantification of adult cutaneous wound volume over time. Data represented as the mean ± SE, n = 6. *p< 0.05.

C57BLKS-Lepr^db/db^ (db/db) diabetic mice and their littermate control C57BLKS-Lepr^db/+^ (db/+) were wounded and studied at 0, 3, 7, 10, and 14 days post-wounding. Matlab program code (**S1C Fig in S1 File**) was written to enhance the visualization of B-mode images. Interestingly, due to acute inflammatory response in the control group, the wound volume increases in the early phase followed by resolution of inflammation and subsequent decrease of the wound volume. This observation has been documented through reports from our group [9]. In contrast, diabetic delayed and sustained inflammation; hence this phenomenon of transient increase in wound volume was not observed. Non-diabetic adult mice (db/+) demonstrated wound closure in 14d post-wounding (**Fig 5A and 5B and S5A Fig in S1 File**). In contrast, wound bed of diabetic mice showed persistent hypercellularity indicative of prolonged inflammation (**Fig 5A and 5B**). This was also verified through histology at d14 (**S5B Fig in S1 File**). The color images provided in Fig 5B are the enhancement of the gray scale signal intensity of the images of Fig 5A. Blue corresponds to low signal and red a higher signal intensity. Wound bed cellularity was calculated from the wound bed signal intensity per unit area from the gray scale images using Matlab code written by us. On day 14, the wounds of diabetic mice remained open (**Fig 5A–5D**). Quantitative signal intensity representing wound bed cellular density was higher in diabetic wounds compared to control db/+ wounds (**Fig 5C**). In 14 days, post-wounding wound volume was nullified in healthy adult mice whereas in diabetics wound remained significantly larger (**Fig 5D**).

### Biomechanical property of adult wound site

**Fig 6:**
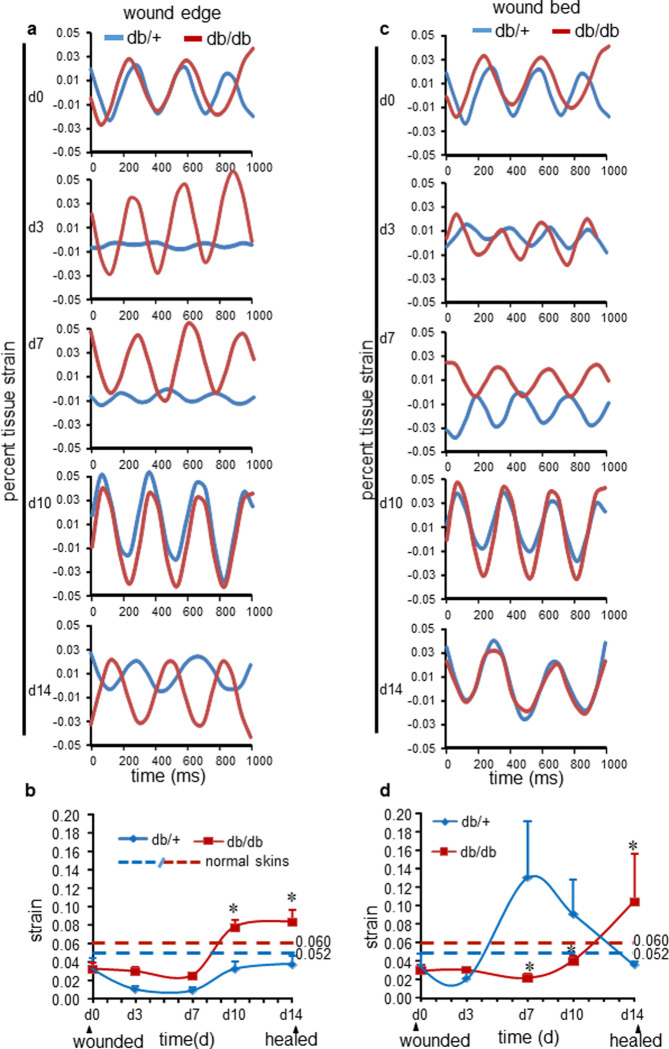
Non-invasive measurement of cutaneous wound tissue elastic-stiffness in adult mice. Ultrasound imaging of adult murine normal skin and stented wounds tissue for the analysis of biomechanical property of db/+ mice (blue) and db/db mice (red). (**a**) Strain curves showing changes in wound edge tissue at 0, 3, 7, 10, and 14d post-wounding of db/+ mice and db/db mice. (**b**) Quantification of strains of normal skin (dotted lines, n = 3), wound edge of db/db and control db/+ mice post-wounding. Data represented as the mean ± SE, *p< 0.05, n = 6. (**c**) Strain curves showing changes in wound bed tissue at 0, 3, 7, 10, and 14d post-wounding of db/+ mice and db/db mice. (**d**) Quantification of normal skin (dotted lines, n = 3), wound bed strain of db/db and control db/+ mice post-wounding. Data represented as the mean ± SE, *p< 0.05, n = 6.

B-mode ultrasound imaging was performed on dorsum of normal healthy and two groups of wounded mice db/db (diabetic) and db/+ (control) over time. Tissue strain was measured from normal skin, wound edge and wound bed (**Fig 6 and S6A Fig in S1 File**). Strain curves from B-mode images of db/+ (blue) and db/db (red) wound-edge tissue are presented from all five time-points from d0 to day 14 post-wounding. (**Fig 6A and 6B)**. Strain curves from B-mode images of db/+ (blue) and db/db (red) wound-edge (**Fig 6A and 6B**) and wound-bed (**Fig 6C and 6D**) tissue is presented from all five time-points from d0 to day 14 post-wounding. Normal skin strain of db/+ (blue) and db/db (red) are asynchronous (**S6B Fig in S1 File**). At d0 post-wounding the control (db/+) and diabetic (db/db) adult wound edge and bed tissue displayed comparable amplitudes. Thus, at this time-point wound-edge tissue stiffness was comparable between non-diabetic and diabetic mice. In this model of excisional wound healing, the inflammatory phase peaks at day 3 and starts to resolve at day 7 post-wounding [37]. At these time points (d3 and d7), strain curves of non-diabetic and diabetic wound edge and bed were asynchronous (**S6B Fig in S1 File**) depicting a direct contrast of biomechanical properties of the post-wound non-diabetic and diabetic adult tissue compartment. The synchronicity in wave pattern indicated that compared to the non-diabetic mice, wound tissue of diabetic mice was heterogeneous in biomechanical properties [17] **(Fig 6A–6D)**. The difference between these two groups was also markedly amplified during the acute-inflammatory phase (d3). On day 3—day 7, the diabetic wound-edge tissue became stiff by acquiring strain in contrast to the non-diabetic wound-edge and normal skin tissue which showed reduced strain **(Fig 6B)**. At d10-d14, wound-edge of control mice acquired transient increased strain which was followed by restoration of elasticity approaching that of the normal skin (dotted blue and red lines) as the wound closes. The pattern of change in the elasticity of wound-edge tissue of diabetics was strikingly different. Severe strain acquired during the early inflammatory phase persisted with a slower recovery of elasticity compared to that of the non-diabetic group (**Fig 6D**). Further long-term study may require concluding about the elasticity of scarring tissue in adult cutaneous wounds.

### Pulse pressure of feeder artery supplying the wound tissue

**Fig 7:**
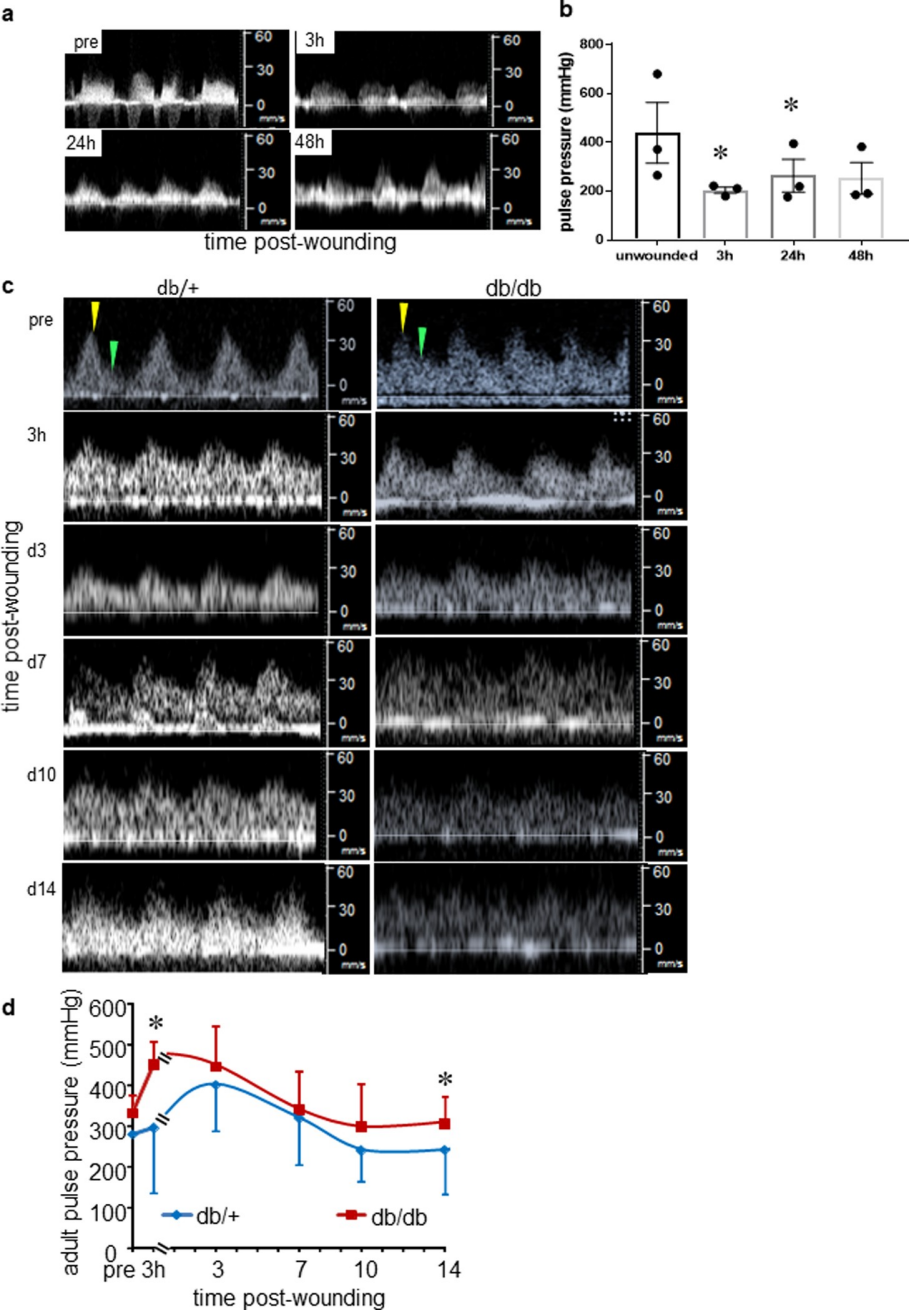
Hemodynamics of fetal and adult cutaneous wound feeder vessels. (**a**) Pulsed Doppler blood flow velocity profile of fetal cutaneous wounds at 3-, 24- and 48-hours post-wounding compared with pre-wounding condition. (**b**) Pulse pressure of feeder vessels unwounded and post-wounding plotted over time. Data presented as the mean ± SE. n = 3, *p< 0.05 compared to 3h. (**c**) Representative Doppler ultrasound images depicting blood flow in feeder vessel of adult cutaneous wound, db/+ (left panel) and db/db (right panel). Systolic peak shown with yellow arrow, diastolic peak shown in green. (**d**) Adult wound feeder artery pulse pressure db/+ (blue) and db/db (red) plotted as line graph over time. The comparison of pulse pressure was made between db/+ (blue line) and db/db (red line) at different time points. Data represented as the mean ± SE. n = 6. *, p< 0.05.

Hemodynamic properties of the blood flow in the artery supplying the wound-site were studied using color Doppler flow imaging. Since fetus get nutrition and oxygen supply from the blood flowing from the uterine arteries of mothers which is demonstrated as a representative color Doppler flow map in S7 Fig in S1 File. The velocity of blood flow at systole and diastole were obtained using pulse wave Doppler technique (**Fig 7A**). Then using the velocity measured at systole and diastole, pulse pressure was calculated using the modified Bernoulli equation [9]. Because the fetal wound closed in 48h, three early time-points were selected for study (**Fig 7A and 7B**). Compared to pulse pressure measured on unwounded healthy skin, 3h of E15.5 fetus, the pulse pressure sharply fell post wounding. Over the subsequent time period, as the wound approached closure, the pulse pressure steadily recovered to achieve pre-wounding levels following closure at 48h (**Fig 7B**). In adult mice, the time period to wound closure was longer and therefore changes in pulse pressure of the primary feeder artery was studied over a longer period of time (d14 post-wounding). In both non-diabetic as well as diabetic mice, the pulse pressure transiently increased post-wounding (prewounding-3h) before returning to the pre-wounding levels by d10 post-wounding (**Fig 7C and 7D**).

## Discussion

Histological quantification of skin and its appendages is widely accepted as standard of practice for the study of wound healing [4, 30]. Although such approach has its own merits, it relies on repeated tissue biopsies [4] and therefore has the potential to alter wound healing process itself. Some of such confounding factors may arise from the development of another wound in a pre-existing wound. Yet others may be contributed by the physical and emotional stress caused by recurring biopsies [38]. Current development in dermatological ultrasound imaging positions it as a powerful non-invasive platform to quantitatively image blood flow, tissue kinetics, tissue stiffness, and its three-dimensional architecture [4, 9, 11, 39, 40]. The purpose of this study is to show that the ultrasonography is feasible in assessing the fetal wound healing in a context of birth related injuries and the fetal wound healing is scarless [25]. In addition, a longitudinal assessment of diabetic adult wounds was also performed in context of changes in biomechanical properties such as stiffness. In our previous study [9], we used ultrasound imaging to study porcine wounds. Though as a model, porcine studies are superior to that of murine, certain parameters (fetal wounding, diabetic conditions) are technically challenging to study in porcine models. In light of that, this study was conducted to investigate assessment of murine fetal cutaneous wound healing and investigate the biomechanical properties of wound healing under diabetic conditions. These addressed new and in-depth advancement to the prior study.

This work reports on the potential of high-resolution multi-modal ultrasound imaging to study multiple aspects of wound healing biology adopting an approach that is of direct value to clinical applications.

In adults, wound healing typically involves a scarring response caused by excessive deposition of connective tissue [41]. The underlying mechanisms are discrepant across fetal and adult systems. In adult wounds, epidermal cells crawl over the exposed substratum to close the wound gap, whereas in the fetus the wound is closed primarily by “purse-string” contraction of rapid assembly of actin filaments [42]. Understanding these differences in healing patterns of fetal and adult wounds using non-invasive imaging modalities will help in better image-guided interventions aimed towards wound healing outcomes. Embryonic wound healing, distinct from adult wound healing, is a subject of major interest because it displays a regenerative scarless phenotype [25, 28, 42]. In this study, ultrasound B-mode imaging enabled quantitative assessment of fetal wound parameters such as wound depth, area and volume. Furthermore, images were reconstructed to enable 3D visualization and quantification. B-mode videos were processed offline to measure biomechanical strain of the fetal skin. Pulse pressure of the feeder blood a vessel at the wound-site was computed using blood flow velocity measured using ultrasound Doppler mode. Non-invasive ultrasound imaging was effective in demonstrating that fetal wounds in mice heal faster and show complete epidermal healing [25, 29, 42, 43] within 48h post-wounding at E15.5. In this study, only wounds of similar location and sizes were used to see the healing pattern. For the transitional mode from E15.5 to E18.5, the wound induced at E15.5 already healed by E18.5. Considering the pregnant mothers’ health condition multiple surgeries was also an issue with respect to humane treatment of experimental animals. The data we present are valuable first evidence from a difficult experimental setting of fetal wound healing. Ultrasound B-mode grayscale signal-based image analysis show signals are affected by a variety of tissue components such as skin pigments, skin layers, adipose, muscle the pathology of the tissue [34, 44, 45]. In addition to that, a number of other factors including power of the input signals, ultrasound transducer response, receiver gain, and imaging algorithms for pre- and post-processing. Previous reports have found that the wound site was observed as a hypoechoic or a non-echoic region [9, 46, 47]. There is a positive co-relation between ultrasound signal intensity and cellular density [36, 44, 48]. The imaging regimen employed with the Matlab code was effective to study tissue cellular density and granulation. Limited wound healing is a major diabetic complication. Whether biomechanical properties of skin is responsible for such impairment remains a topic of active investigation [37, 49]. This work provides insight by comparing the tissue cellularity between diabetic and non-diabetic wounds. Diabetic cutaneous wound tissue displayed more cellularity indicative of enhanced inflammation and fibrosis [50] during the time course of healing. Poor biomechanical properties such as augmented tissue stiffness and reduced elasticity are known to be caused by persistent inflammation and fibrosis [17, 37, 39, 51, 52]. Wound dehiscence and scarring are two major sequences of abnormal wound healing. Measuring the biomechanical properties of the wounds during the healing course could be a good indicator of the healing quality. It helps with interpreting and understanding the cellular changes during the wound healing process. Wound healing is a dynamic process that consists of waves of cellular changes, starting with the inflammatory cells that will initiate the wound healing followed by the proliferative phase with significant contribution of fibroblasts and finally the remodeling phase [46, 47]. Any cellular change will affect the biomechanical properties of the skin. For example, in acute wound the biomechanical properties are restored similar to normal skin post-healing. However, in chronic wound conditions such as with prolonged inflammation will result in partial restoration of biomechanical properties and consequently the healed wound will be prone to dehiscence [3, 46]. In contrast, the abnormal wound healing associated with keloid and fibrosis in which the excessive deposition of collagen and extracellular matrix from fibroblast result in changes of the biomechanical properties with decreasing the skin elastic modulus and increasing the stiffness of the healed skin compared to normal skin [36, 53–55]. Biomechanical properties can be assessed non-invasively through ultrasound imaging which can allow the evaluation of changes in the dermis and also enable therapeutic intervention to be assessed [56–58].

The biomechanical properties of strain and elasticity in correlated to tissue cellularity and granularity. Thus, study of these biomechanical property reflects the wound healing process. Wound healing is impaired in diabetic animal model. The study investigates this impaired healing in db/db animals in the context of biomechanical properties. Currently, there are different testing protocols to assess tissue biomechanical properties making data interpretation from different studies difficult [5, 30]. However, in agreement with the previous studies on human tissue samples [59] this work found that an inverse relationship between cell density and tissue stiffness at low deformation levels as assessed by non-invasive ultrasound imaging [13, 45]. In diabetic mice, severe strain acquired during the early phase of wound healing was followed by slower recovery of elasticity during the later stage compared to that of the non-diabetic mice. The ultrasound platform utilized in the present study was effective in studying wound-site hemodynamics in both fetal and adult mice using Doppler flow technique employed on gated feeder arteries [20]. The pulse pressure generated in the fetal blood flow was governed by the elastic properties of the blood vessels and not by the pulse pressure generated by the mother’s beating heart [60]. This work constitutes maiden report presenting a non-invasive method to quantify the elastic strain of the cutaneous wound tissue of fetuses and that of diabetic and non-diabetic wounds of adult mice. While stiffening of the repairing skin is transient in non-diabetics [17], such change was slow to mount but persisted in diabetics. Whether such difference is contributed by persistent inflammation in diabetics emerge as a reasonable question.

Clinically, ultrasound technology is readily available to monitor fetal development and to intervene in case of developmental anomalies [42, 60, 61]. Obstetric use of ultrasound commonly identifies the need and guides fetal surgery. Ultrasound serves to guide real-time intraoperative monitoring, precise intervention and minimizing the hazards of hysterotomy [14, 62]. This work demonstrates the versatility of a novel ultrasound imaging platform in non-invasively probing functional aspects of the healing wound in fetal and adult conditions. Importantly, the imaging technology is applicable clinically and may be viewed as a viable addition to wound care.

## Supporting information

S1 File (PDF)

S1 Video (ZIP)
