# Supplementary material for: High resolution ultrasound imaging for repeated measure of wound tissue morphometry, biomechanics and hemodynamics under fetal, adult and diabetic conditions
Source: PLoS One. 2020 Nov 23;15(11):e0241831. doi: 10.1371/journal.pone.0241831 (PMC7682876; doi:10.1371/journal.pone.0241831)
Supplement: S1 File — (PDF) [file pone.0241831.s001.pdf]

High Resolution Ultrasound Imaging for Repeated Measure of Wound Tissue Morphometry, Biomechanics and Hemodynamics under Fetal, Adult and Diabetic Conditions

Surya C Gnyawali, PhD[2], Mithun Sinha, PhD[1,2], Mohamed S El Masry, MD[1,2,4], Brian Wulff, PhD[3], Subhadip Ghatak, PhD[1,2], Fidel Soto-Gonzalez, BS[2], Traci A Wilgus, PhD[3], Sashwati Roy, PhD[1,2], Chandan K Sen, PhD*[1,2]

[1]Department of Surgery, IUH Comprehensive Wound Center, Indiana Center for Regenerative Medicine and Engineering, Indiana University School of Medicine, Indianapolis, IN 46202, USA
[2]Department of Surgery, Davis Heart and Lung Research Institute, Center for Regenerative Medicine & Cell-Based Therapies, The Ohio State University Wexner Medical Center, Columbus, OH 43210, USA
[3]Department of Pathology, The Ohio State University Wexner Medical Center, Columbus, OH 43210, USA
[4] Department of Plastic and Reconstructive Surgery, Zagazig University, 44519, Egypt

***Corresponding Author:** Chandan K. Sen, PhD
975 W Walnut St,
Medical Research Library Building, Suite 454
Indiana University School of Medicine
Indianapolis, IN 46202.
Tel. 317 278 2636
E-mail: cksen@iu.edu

Running Title: *Non-invasive characterization of fetal and adult wounds*

Supplementary Figure Legends

Supplementary Fig S1a. Enlarged Doppler color flow images (from **Fig 1a**) of fetus at days (i) E6.5, (ii) E13.5 and (iii) E15.5 are shown. Scale bar = 5 mm

Supplementary Fig S1b. Flow chart of image processing and strain analysis.

Supplementary Fig S1c. Matlab code for enhancement of tissue density.

Supplementary Fig S2. Representative three-dimensional images of fetal wounds. (a) Enlarged view of Fig 2a. (b-d) 3D reconstructed images of the fetus at 3, 24, and 48 hours post-wounding. Fetal wounds sites are shown in cyan.

Supplementary Fig S5a. Morphometry of adult diabetic (db/db) cutaneous wound healing compared to the non-diabetic (db/+) mice d14 post-wounding in six different mice (i-vi) in each group. Grey: ultrasound B-mode image of full thickness stented wound bed (red arrow, white dotted region), wound edge (yellow arrows) of db/db and control db/+ mice. Color: enhanced anatomical images using Matlab code to show the cellular density. Density index low (blue) to high (red)

Supplementary Fig S5b. Histology of db/+ and db/db wounds at d14 post-wounding. Hematoxylin and Eosin staining. Scale bar = 500 µm for the mosaic containing the entire section. Scale bar = 100 µm for zoomed inset.

Supplementary Fig S6a. Adult wound tissue site for biomechanical strain measurement. A green line was traced around the wound site to help select wound edge and bed for strain analysis using ‘VevoStrain®’ software. Selected blue dot (i) for adult wound edge and (ii) wound bed to analyze biomechanical strain.

Supplementary Fig S6b. A green line was traced around the normal skin/wound site to help select skin tissue strain analysis using ‘VevoStrain®’. Adult tissue strain curves for biomechanical elastic strain patterns and synchronicity. Blue curve for db/+ and red curve for db/db (i). Wound strain synchronicity curves over time of wound edge (ii) wound bed (iii) to analyze biomechanical strain.

Supplementary Fig S7. Color Doppler flow images of uterine artery imaged at E15.5. The uterine artery as indicated by white arrow shows blood flowing across the placenta to the fetal heart.

Supplementary Videos. SV1 Ultrasound images of embryos at days (a) E6.5, (b) E13.5, and (c) E15.5

Supplementary Videos. SV2 (a) Representative three-dimensional reconstruction image of fetal wounds. (bd) 3D reconstructed images of the fetal wound at (b) 3, (c) 24, and (d) 48 hours post-wounding. Fetal wounds sites are shown by arrow.

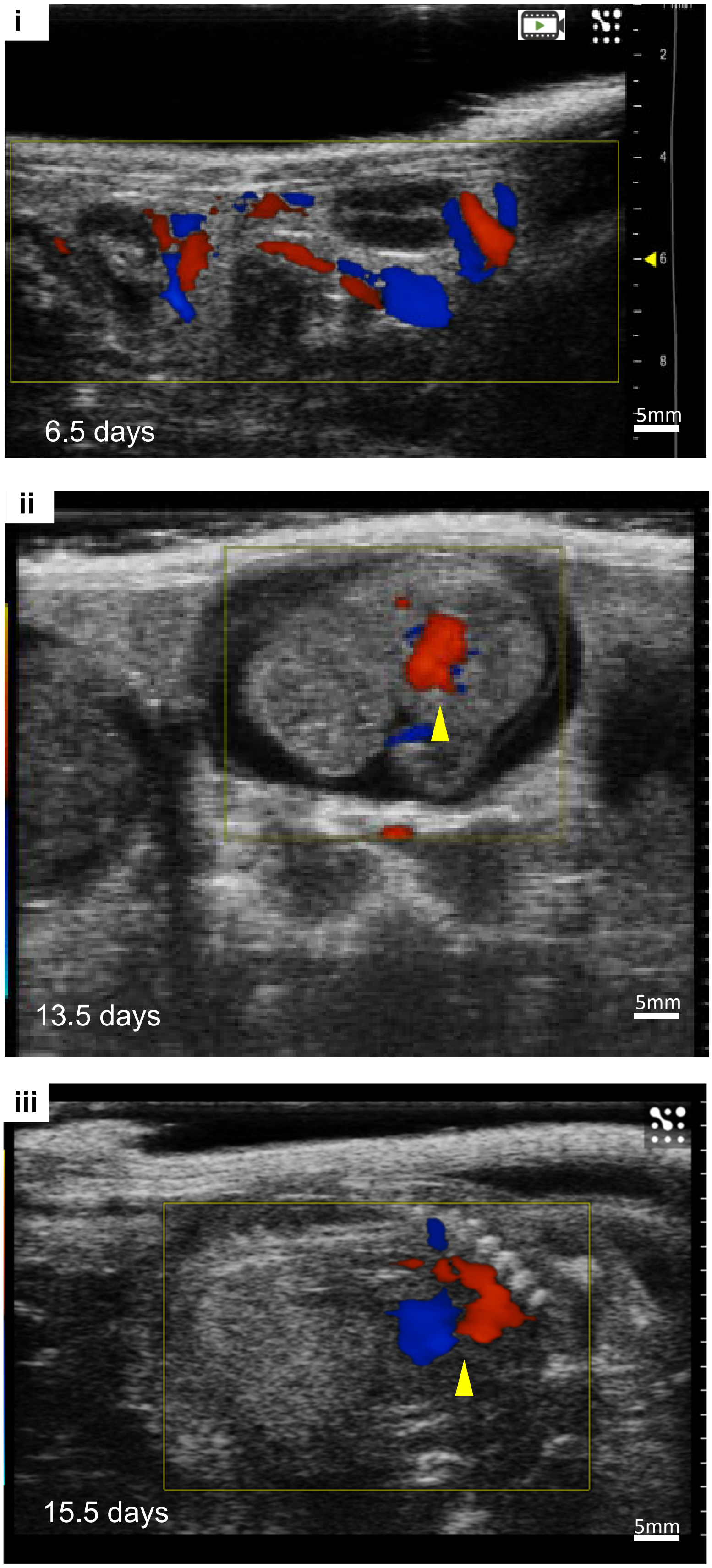

Supplementary Fig. S1a

Flow chart: design of image processing and strain analysis

Image processing

Export Ultrasound images

Offline processing, VevoLab software

Measure vascular sizes, skin thickness, blood flow

Compute volumes, cell density using Matlab code based on signal intensity, Calculate Pulse pressures using velocities at systole and diastole on Bernoulli's equation

Fetal/Adult strain analyses

From the B-mode videos, Locate fetuses & wound sites

Draw a green line around the wound site to select normal skin, wound edge and bed for strain analysis using 'VevoStrain' software. Select a dot on the traced green line, blue for skin, red for wound edge and green for wound bed to get respective strain values.

From the strain values correlate stiffness

From the strain curves compare the synchronicity through the phase match/mismatch

Figure S1b

```
Clc;
clear all;
close all;
im = imread('fetus1.jpg');
I = rgb2gray(im);
figure;
imshow(im);
rgbImage = ind2rgb(I, jet(256));
imshow(rgbImage);
maskedImage  = double(repmat(rgbImage, 1, 1, 3));
imshow(maskedImage);
% Enlarge figure to full screen.
set(gcf, 'Units', 'Normalized', 'OuterPosition', [0 0 1 1]);
%---------------------------------------------
%code below changes the pixels to green
Image1=im2double(rgbImage);

[row column page] = size(Image1)

mask = Image1(:,:,2) > Image1(:,:,1) & Image1(:,:,2) > Image1(:,:,3);

Image2 = Image1 .* mask(:,:,[1 1 1]);

figure,imshow(Image2);

title('light green');

mask = Image2(:,:,2) + 50 > Image2(:,:,1) & Image2(:,:,2) + 50 >
Image2(:,:,3);

Image3 = Image2 .* mask(:,:,[1 1 1]);

figure,imshow(Image3);

title('dark green');
```

Supplementary Fig. S1c

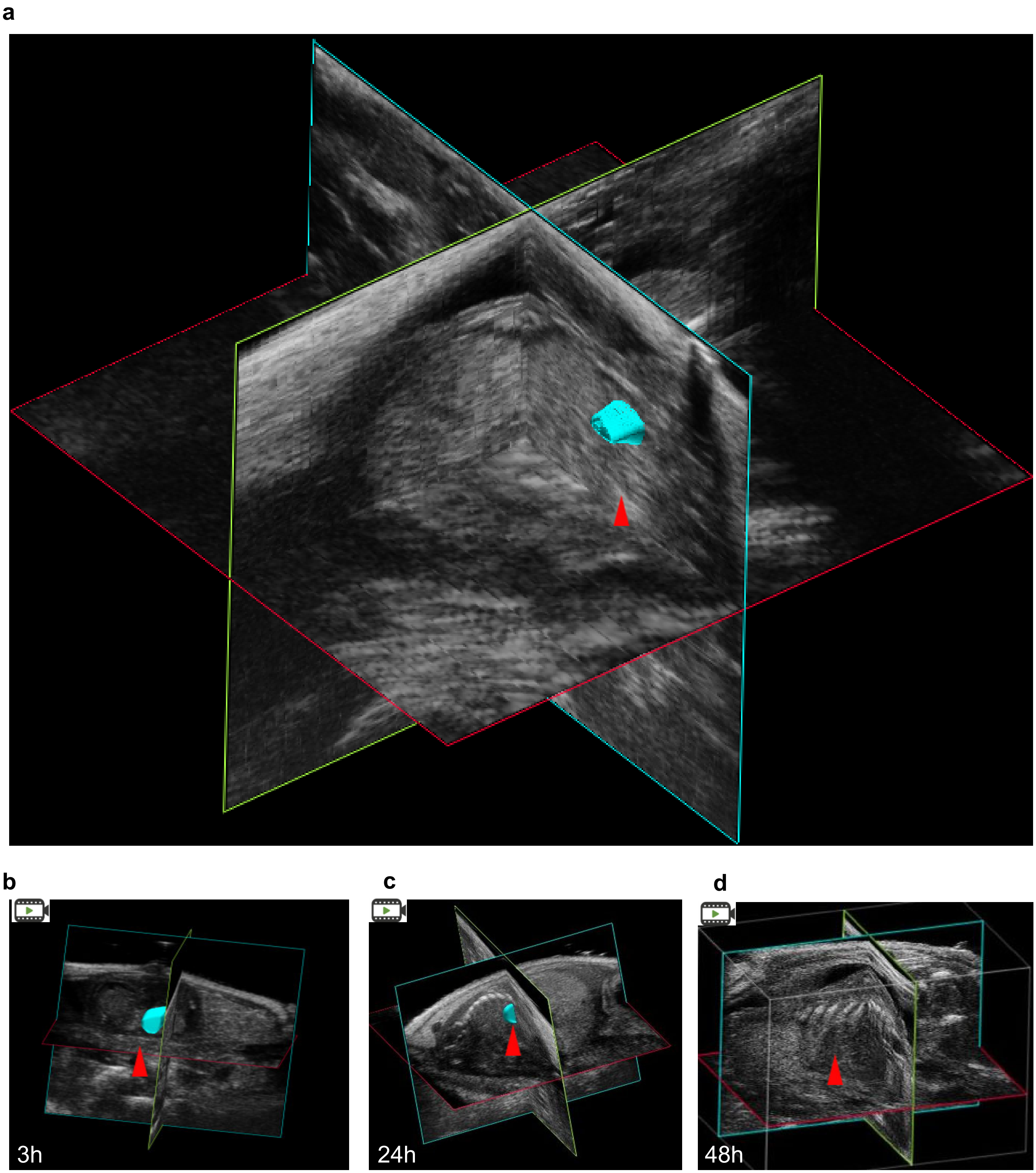

Supplementary Fig. S2

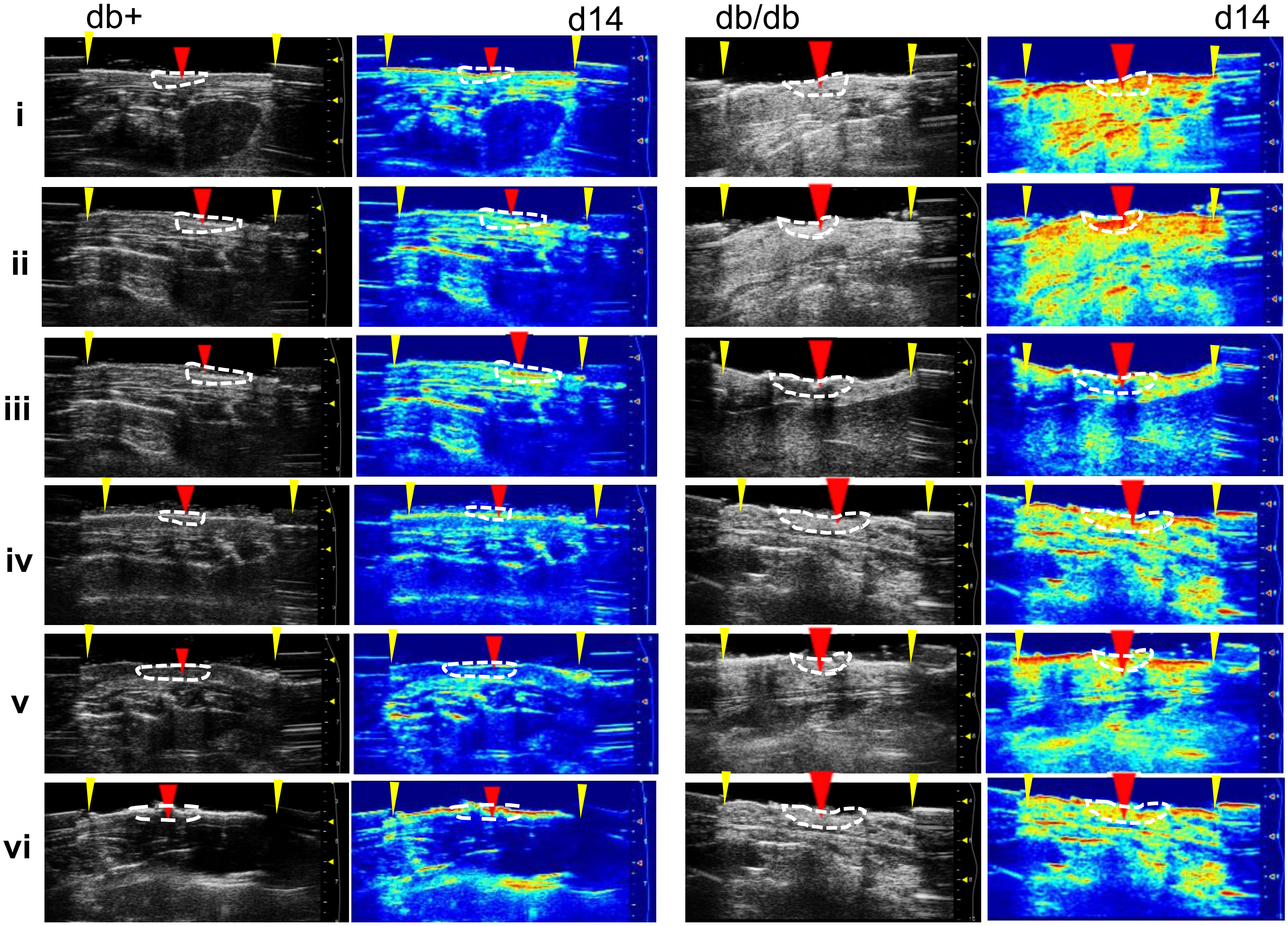

Figure S5a

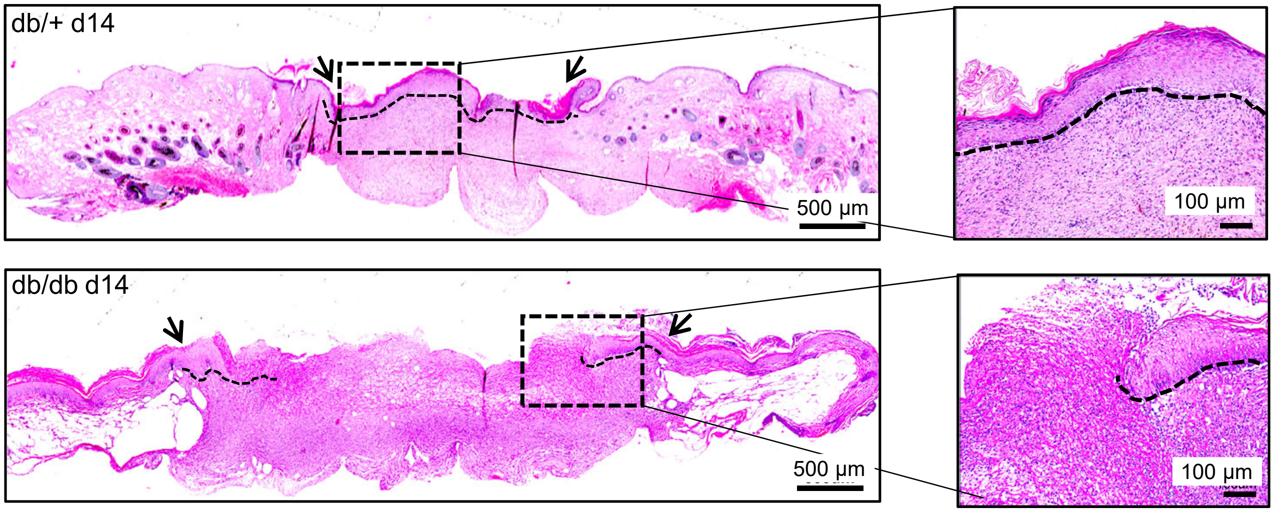

Figure S5b

i

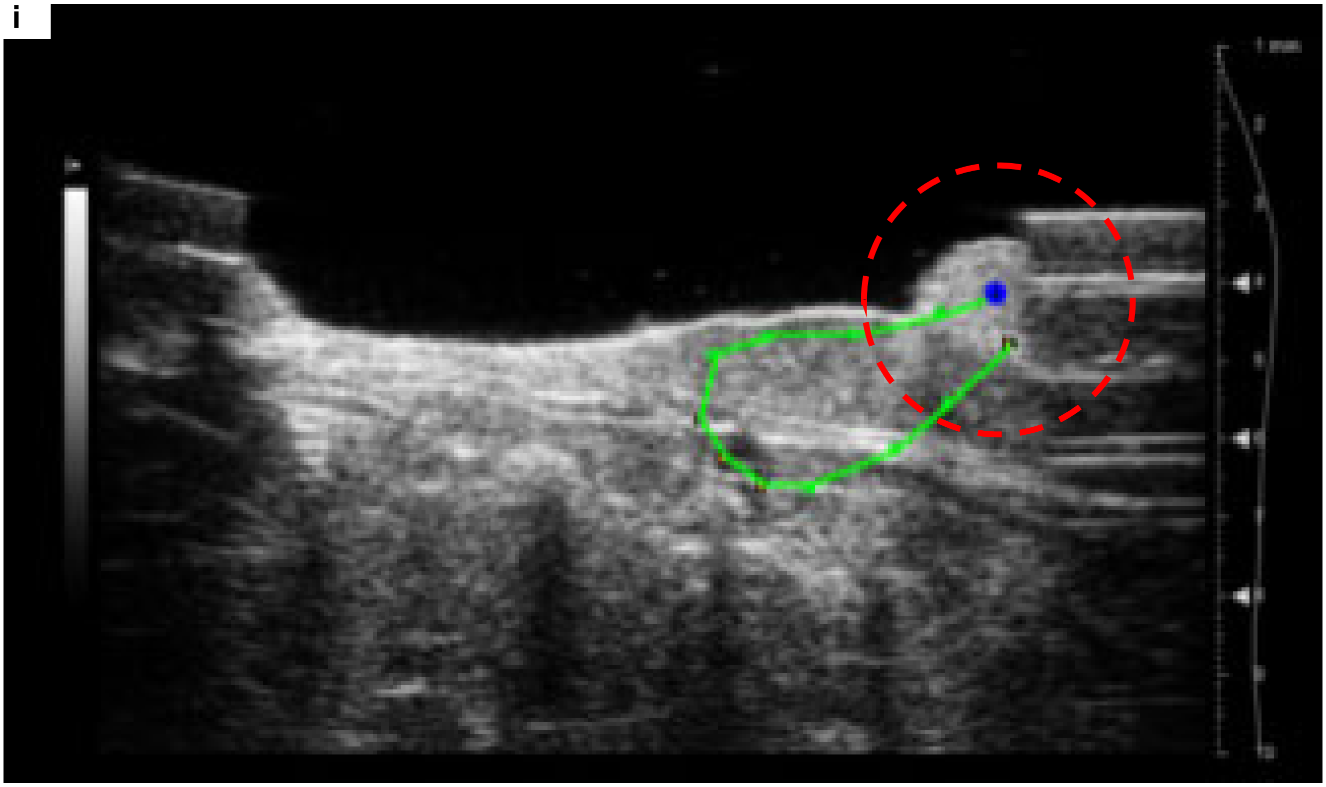

ii

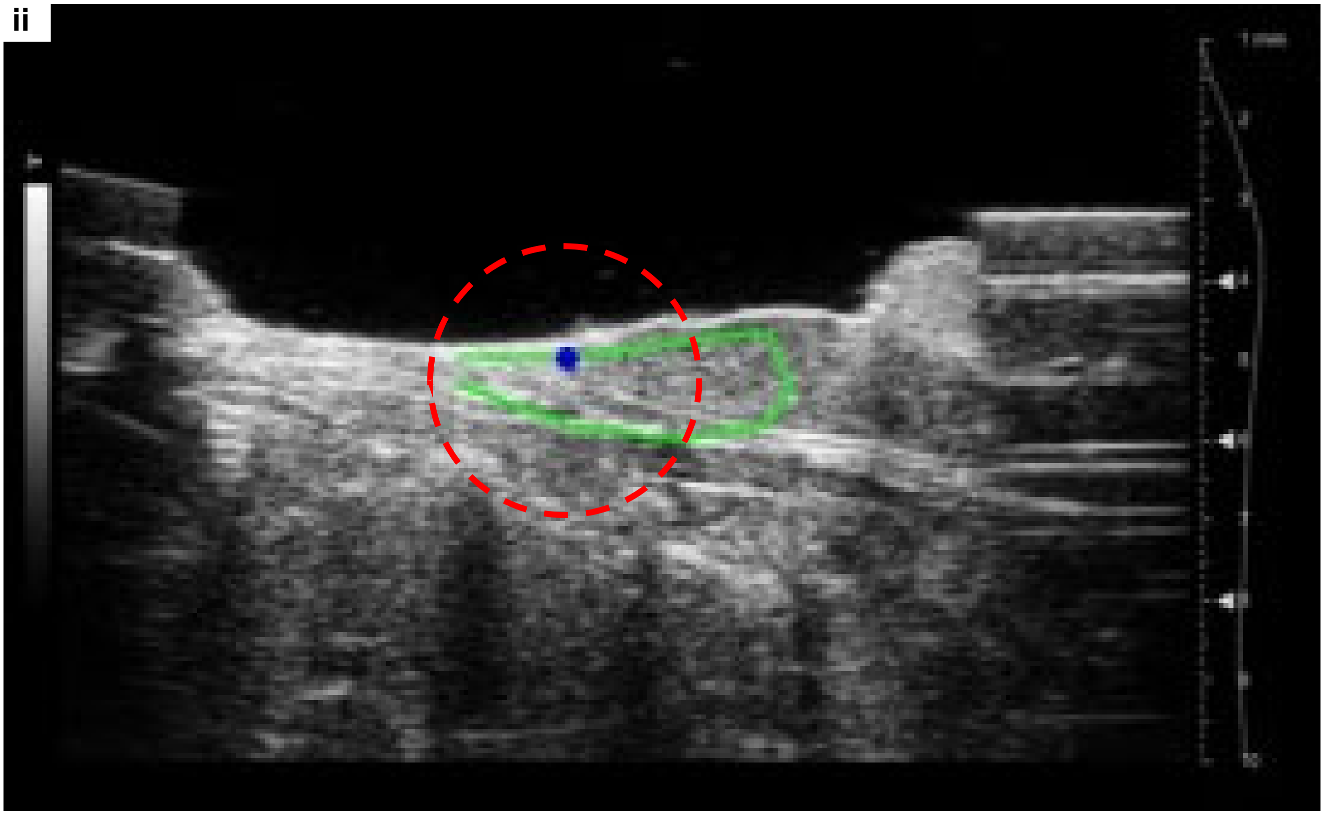

Figure S6a

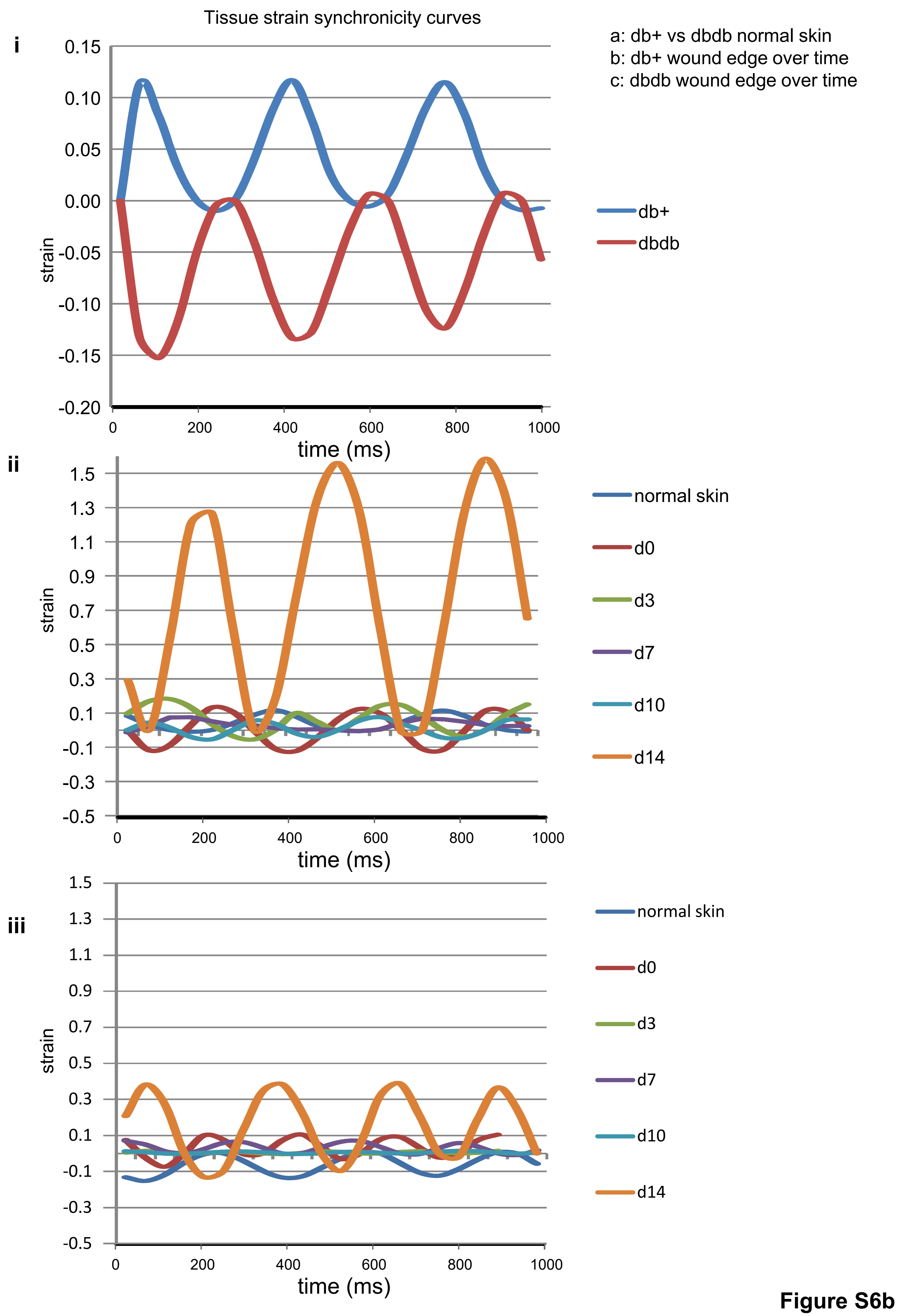
Tissue strain synchronicity curves
a: db+ vs dbdb normal skin
b: db+ wound edge over time
c: dbdb wound edge over time
i
0.15
0.10
0.05
0.00
-0.05
-0.10
-0.15
-0.20
strain
0
200
400
600
800
1000
time (ms)
db+
dbdb
ii
1.5
1.3
1.1
0.9
0.7
0.5
0.3
0.1
-0.1
-0.3
-0.5
strain
0
200
400
600
800
1000
time (ms)
normal skin
d0
d3
d7
d10
d14
iii
1.5
1.3
1.1
0.9
0.7
0.5
0.3
0.1
-0.1
-0.3
-0.5
strain
0
200
400
600
800
1000
time (ms)
normal skin
d0
d3
d7
d10
d14

Figure S6b

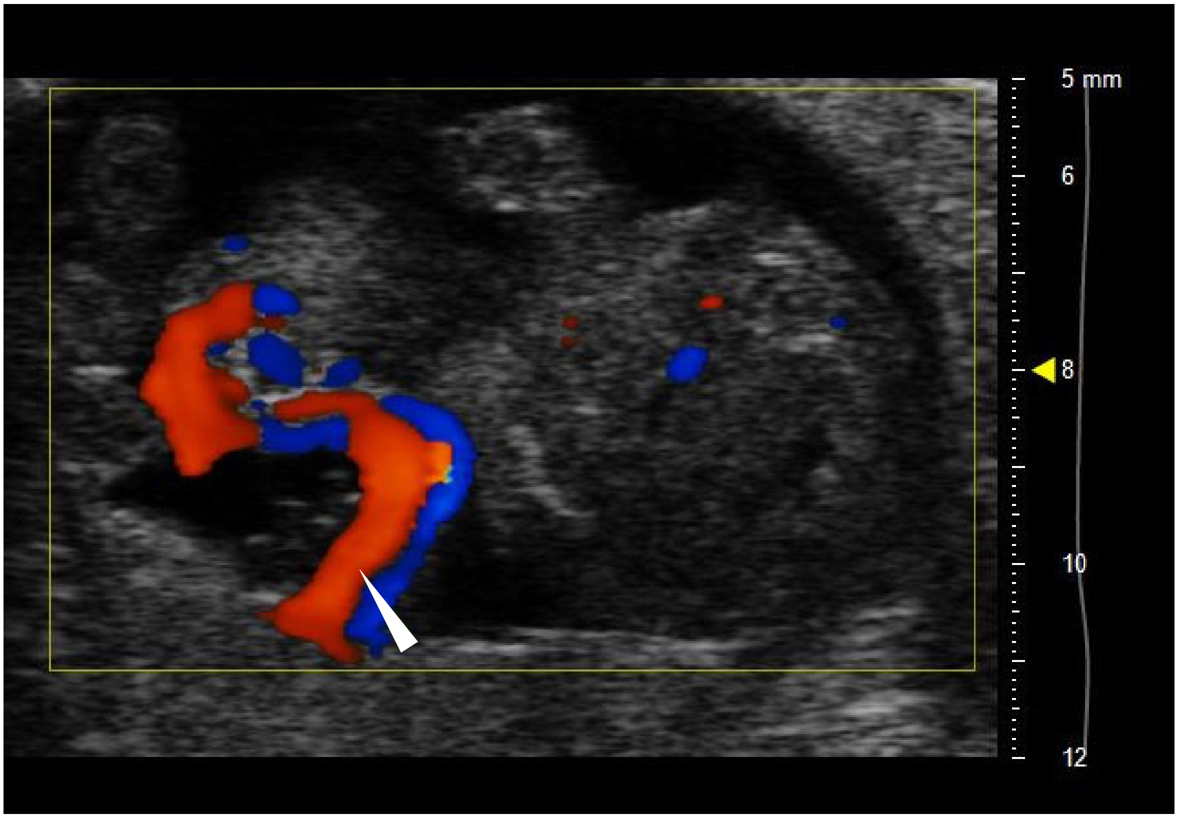

Figure S7
